# Zn Promoted Mg-Al Mixed Oxides-Supported Gold Nanoclusters for Direct Oxidative Esterification of Aldehyde to Ester

**DOI:** 10.3390/ijms22168668

**Published:** 2021-08-12

**Authors:** Jie Li, Shiyi Wang, Huayin Li, Yuan Tan, Yunjie Ding

**Affiliations:** 1 Hangzhou Institute of Advanced Studies, Zhejiang Normal University, 1108 Gengwen Road, Hangzhou 311231, China; lijie_zjnu@163.com (J.L.); wangshiyi@dicp.ac.cn (S.W.); lihuayin@zjnu.edu.cn (H.L.); 2 Dalian National Laboratory for Clean Energy, Dalian Institute of Chemical Physics, Chinese Academy of Sciences, Dalian 116023, China; 3 The State Key Laboratory of Catalysis, Dalian Institute of Chemical Physics, Chinese Academy of Sciences, Dalian 116023, China

**Keywords:** gold catalyst, cluster, hydrotalcite, oxidative esterification, methyl isobutyrate

## Abstract

The synthesis of ester compounds is one of the most important chemical processes. In this work, Zn-Mg-Al mixed oxides with different Zn^2+^/Mg^2+^ molar ratios were prepared via co-precipitation method and supported gold nanoclusters to study the direct oxidative esterification of aldehyde and alcohol in the presence of molecular oxygen. Various characterization techniques such as N_2_-physical adsorption, X-ray diffraction (XRD), transmission electron microscopy (TEM), X-ray photoelectron spectroscopy (XPS) and CO_2_ temperature programmed desorption (TPD) were utilized to analyze the structural and electronic properties. Based on the results, the presence of small amounts of Zn^2+^ ions (~5 wt.%) provoked a remarkable modification of the binary Mg-Al system, which enhanced the interaction between gold with the support and reduced the particle size of gold. For oxidative esterification reaction, the Au_25_/Zn_0.05_MgAl-400 catalyst showed the best performance, with the highest turnover frequency (TOF) of 1933 h^−1^. The active center was believed to be located at the interface between metallic gold with the support, where basic sites contribute a lot to transformation of the substrate.

## 1. Introduction

Ester compounds are one of the most important raw materials in chemical industry and organic synthesis and constitute numerous artificial fragrances, flavoring agents, solvent extractants and chemical intermediates [1]. The traditional synthetic method for preparation of esters involves a two-step procedure, including the oxidation of aldehyde and/or alcohol and the subsequent esterification between carboxylic acid and its activated derivatives with alcohols [2,3,4]. The multistep process involves the production of large amounts of toxic wastes and irremovable byproducts, which is not conducive to the development of green chemistry. Therefore, the synthesis of esters through one-step oxidative esterification from aldehydes and alcohols has attracted great interest in recent years.

Traditionally, the direct oxidative esterification requires homogeneous stoichiometric reagents, such as KMnO_4_ [5], CrO_3_ [6], H_2_O_2_ [7], etc., while the strong oxidants not only call for harsh conditions but also cause separation difficulties. Therefore, the green oxidants such as air and dioxygen over heterogeneous catalysts under mild conditions are highly desirable. The supported noble metal Pd-based catalysts have been reported to be efficient for one-step oxidative cross-esterification reaction between aldehyde and alcohol in the presence of molecular oxygen [8,9,10,11,12]. However, the selectivity of the corresponding esters is typically low and this process requires the addition of environmentally unfriendly heavy metals, such as Pb and Bi [8,9,10]. Furthermore, the utilization of liquid base additives is needed for Pd-based catalysts, which makes the process less green and not cost-effective [11,12].

Supported gold catalysts are a great promising candidate for this kind of reaction due to their unique selectivity under mild conditions [13,14,15,16,17,18]. Various transition-metal-oxides and mixed oxides were explored as supports for synthesis of the supported gold catalysts [15,16,17,18]. Nevertheless, their catalytic performances were largely dependent on the particle size of gold [15], the interaction between gold and the carrier [16], as well as the acidic-basic properties of the supports [15,17]. Thus, preparation of highly efficient gold catalysts for synthesis of the corresponding esters is always considered in combination of the size of gold and the properties of the supports.

Mg-Al hydrotalcite (HT) is a common solid base with unique layered structure, which plays an important role in many base-catalyzed reactions, such as alcohol oxidation [19,20], aldol condensation [21] and transesterification [22]. Their catalytic performances can be modified by incorporating various metal ions, such as Cu^2+^ [23,24], Zn^2+^ [25,26], Ni^2+^ [27], Y^3+^ [28], Zr^4+^ [29], etc. For example, Pavel et al. [28] reported yttrium-modified Mg-Al-HTs for epoxidation of styrene with hydrogen peroxide. An increment in catalytic activity was observed with the addition of Y^3+^ cations, which increased the alkalinity of catalysts accordingly. Willinton et al. [25] prepared Mg-Zn-Al HTs and their derived mixed oxides for the aldol condensation reaction. They found the presence of Zn^2+^ ions caused a clear influence on the acidic–basic properties of the catalysts when compared to the binary Mg-Al system, which influenced the activity and selectivity greatly. 

Previously, our group reported that the Zn-Al HT-derived mixed oxides-supported gold catalysts were highly selective and resistant for high temperature sintering in hydrogenation reactions of functionalized nitroarenes due to the strong interaction between gold with the support [30]. Later, we found that the catalyst is also active and selective for synthesis of methyl methacrylate from methacrolein and methanol [31]. Herein, in this work, we try to incorporate Zn^2+^ ions into Mg-Al HTs for synthesis of stable gold catalysts for inorganic base-free oxidative esterification of aldehyde to ester. By adjusting the molar ratios of Mg^2+^/Zn^2+^ ions, the textural and electronic properties of supported gold catalysts were investigated in detail, in which N_2_ physical adsorption-desorption, X-ray powder diffraction (XRD), transmission electron microscopy (TEM), X-ray photoelectron spectroscopy (XPS) and CO_2_ temperature programmed desorption (TPD) were successively explored. Based on these results, a clear insight into the structure-performance relationship over supported gold catalysts for direct oxidative esterification was provided. 

## 2. Results 

### 2.1. Compositional and Textural Analysis of the Catalysts

For synthesis of the Zn-Mg-Al mixed oxides-supported gold catalysts, Au_25_ nanoclusters were firstly prepared through the sol-gel method with cysteine as the protective ligand, defined by UV-visible spectra (Figure S1). Then, different supports with various molar ratios of Zn^2+^/Mg^2+^ were impregnated with the solution of Au_25_. Before each test, the samples were dried overnight and pretreated in muffle at 400 °C for 2 h to remove most of the protective ligands. After that, various characterizations of the structural and electronic properties of the catalysts were carried out under specific conditions. The actual metal loadings of gold were measured by inductively coupled plasma atomic emission spectroscopy (ICP-AES), from which all catalysts showed similar values of ~1.5 wt.% (Table 1), which agrees well with the nominal values.

**Figure 1:**
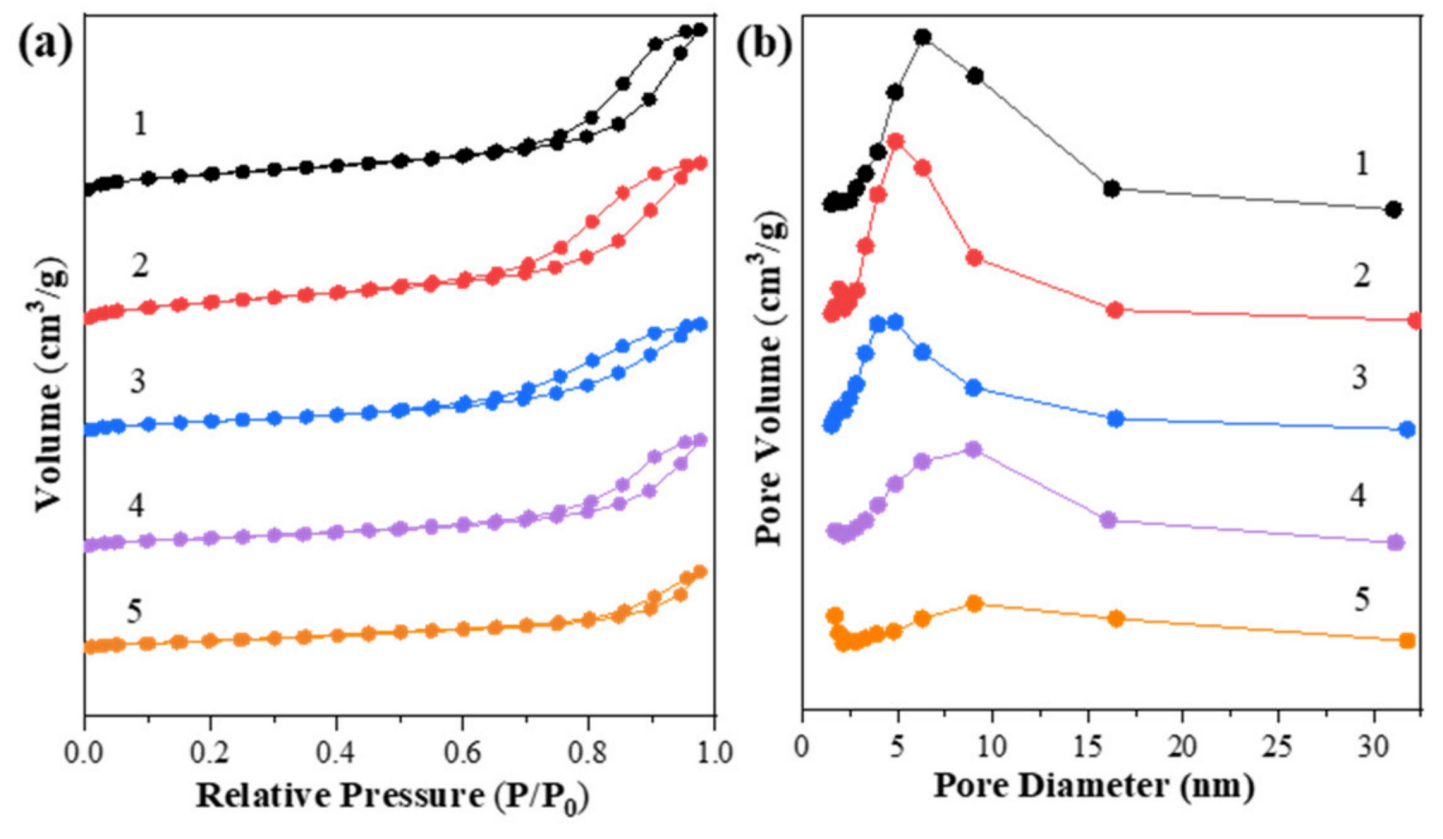
(**a**) N_2_ physical adsorption–desorption isotherms and (**b**) BJH-pore size distributions of Zn-Mg-Al mixed oxides-supported gold catalysts: (1) Au_25_/MgAl-400; (2) Au_25_/Zn_0.05_MgAl-400; (3) Au_25_/Zn_0.33_MgAl-400; (4) Au_25_/Zn_3_MgAl-400; (5) Au_25_/ZnAl-400.

The specific surface area, pore width and pore volume were measured by a nitrogen adsorption–desorption test. The isothermal curves and contrastive results are shown in Figure 1 and Table 1. In Figure 1a, Zn-Mg-Al mixed oxides-supported gold catalysts present typically type IV isotherms according to the IUPAC classification [32]. Furthermore, the hysteresis loops are H3 type and are located at higher relative pressures, indicating the formation of slit-shaped holes and the presence of mesopores, which were derived from the collapse of hydrotalcite structure after calcination [33]. To be noted, with the increase of Zn^2+^ ions into the Mg-Al mixed oxides, the pore volumes and pore sizes of catalysts declined, while the surface area of catalysts increased initially from 221.1 to 242.0 m^2^/g and then decreased drastically to about 100 m^2^/g. This is likely derived from the variation of phase structure of catalysts and it implies that the structural property of catalysts could be adjusted by the addition of Zn^2+^ ions. Later, the structural features were further analyzed by the following XRD results.

### 2.2. Structural Analysis of the Catalysts 

**Figure 2:**
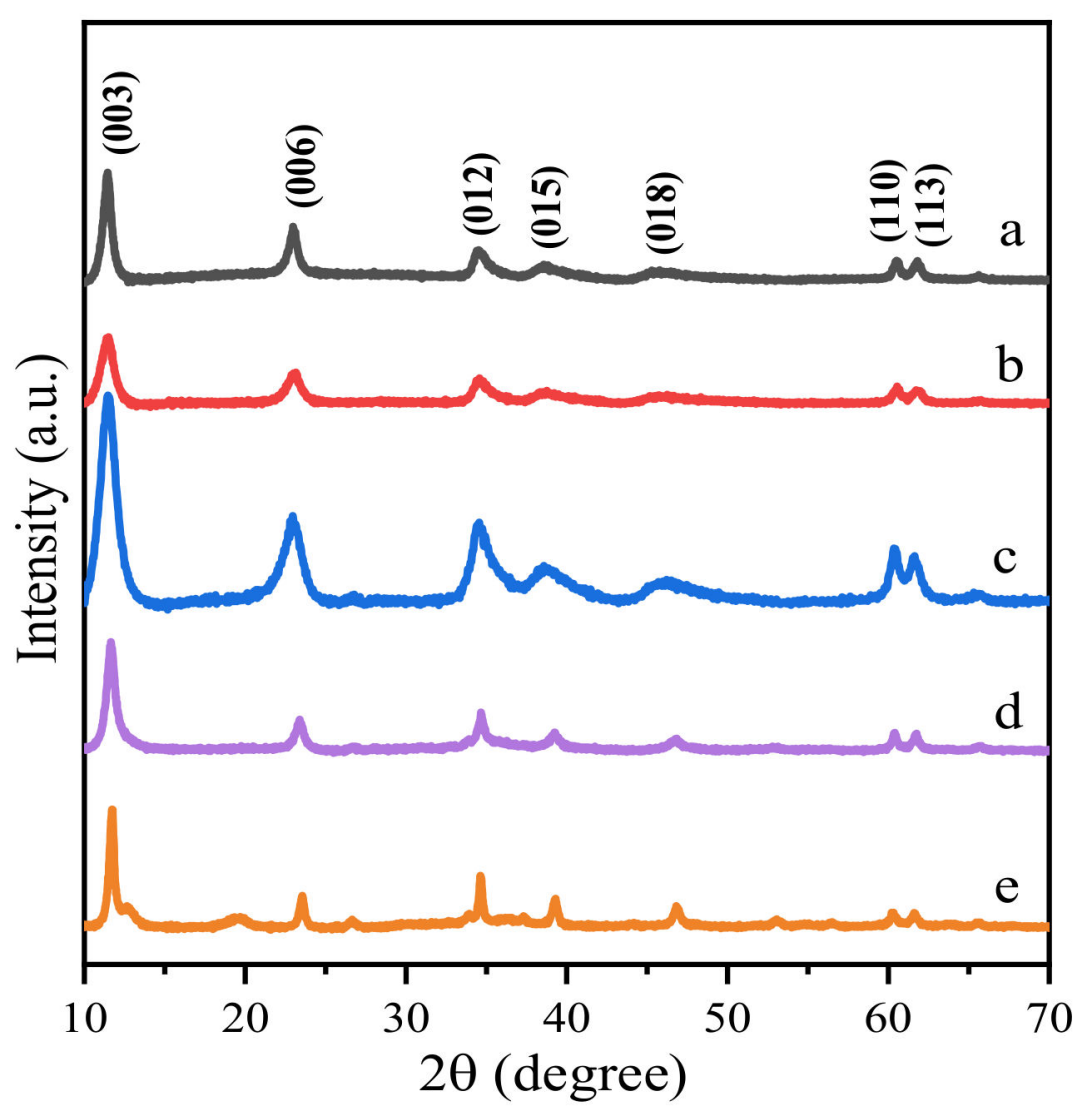
XRD spectra of Zn-Mg-Al HT supported gold nanoclusters: (**a**) Au_25_/MgAl-HT; (**b**) Au_25_/Zn_0.05_MgAl-HT; (**c**) Au_25_/Zn_0.33_MgAl-HT; (**d**) Au_25_/Zn_3_MgAl-HT; (**e**) Au_25_/ZnAl-HT.

**Figure 3:**
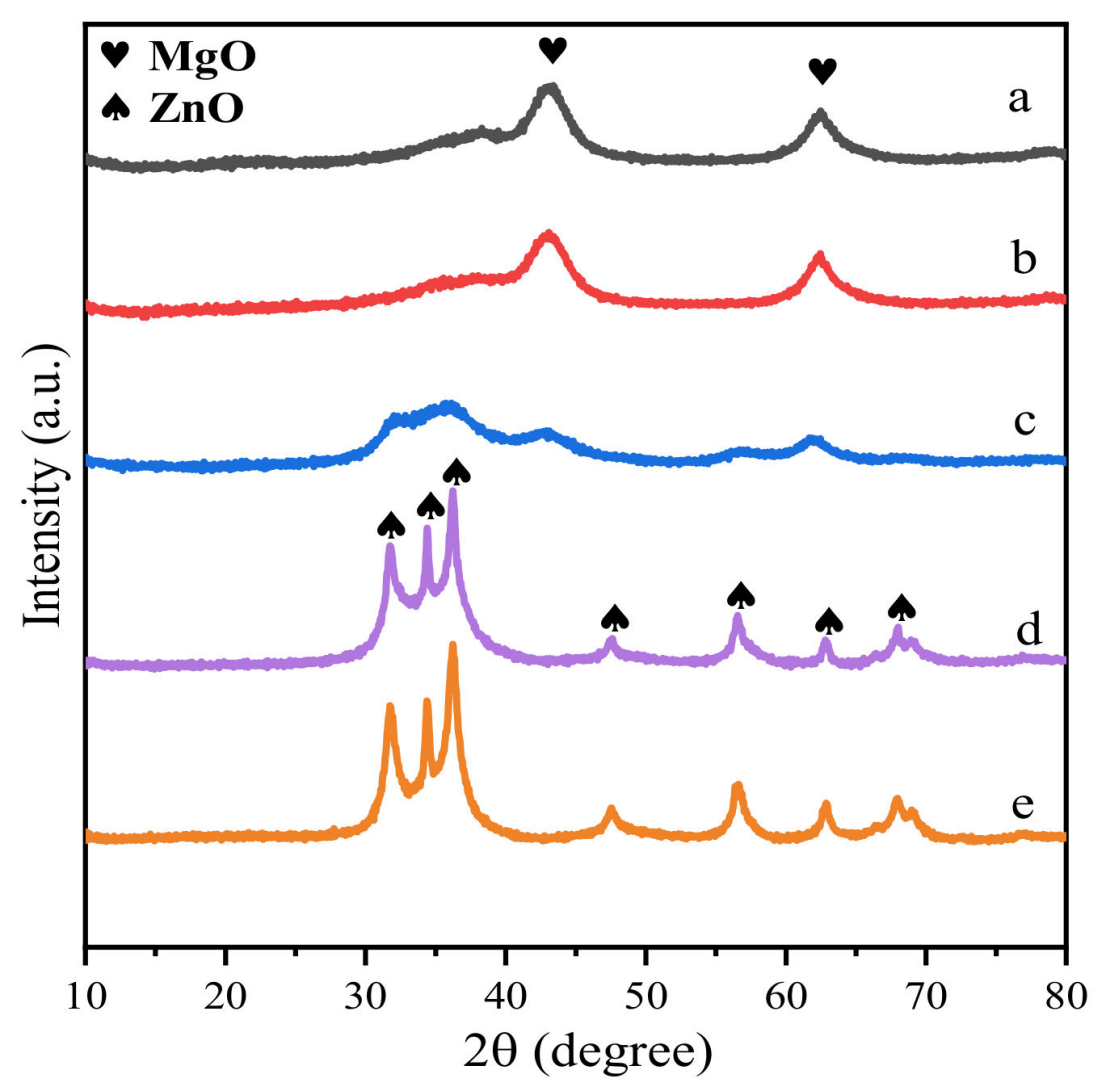
XRD spectra of Zn-Mg-Al mixed oxides-supported gold catalysts: (**a**) Au_25_/MgAl-400; (**b**) Au_25_/Zn_0.05_MgAl-400; (**c**) Au_25_/Zn_0.33_MgAl-400; (**d**) Au_25_/Zn_3_MgAl-400; (**e**) Au_25_/ZnAl-400.

The phase structure of Zn-modified Mg-Al-HT supported gold catalysts before and after thermal treatment was analyzed by X-ray diffraction (XRD). Figure 2 and Figure 3 display the sample diffraction peaks. Before calcination, all samples exhibited layered characteristics of hydrotalcites, with sharp and intense lines located at 11.3°, 22.8°, 34.7°, 39.1°, 46.4°, 60.6° and 61.9° (Figure 2), corresponding to the (003), (006), (012), (015), (018), (110) and (113) planes (standard magnesium aluminum hydrotalcite JCPD NO. 00-035-0965, standard zinc aluminum hydrotalcite JCPD NO. 00-048-1023), respectively. The XRD patterns of the samples with fewer Zn^2+^ ions have broader reflections, which are close to that of the Au_25_/MgAl-HT (Figure 2a), while the samples with more Zn^2+^ content present sharper reflections that are similar to the Au_25_/ZnAl-HT (Figure 2e).

According to the literature, characteristics of (003) and (110) reflections correspond to basal and in-plane spacing [33,34]. Here, the lattice parameters (a and c) in Table 2 were calculated on the basis of a rhombohedral R3m space group and hexagonal cell by the formula of a = 2 d_110_ and c = 3 d_003_ [33,34]. The parameter a represents the average distance between metal and metal in each layer of the hydrotalcite, whereas the parameter c reveals the distance between layer and layer [33]. From the results, the value of a remains constant upon zinc addition, while the value of c gradually decreases with increasing of Zn^2+^. This may be due to the similar ionic radius of octahedral coordinated Mg^2+^(0.72 Å) with Zn^2+^(0.74 Å) [35]. Hence, the effect of Zn substitution on the value of a was negligible. However, the larger electronegativity of Zn (1.66) compared to Mg (1.29) would lead to an increase in layer charge density, which is directly related to the decrease of interlayer distance [36]. Table 2 presents the crystal sizes of different samples that were calculated using Scherrer’s equation. The increased crystallite sizes were consistent with the textural analysis. That is, a higher degree of crystallinity suggests a loss in surface area [36].

Furthermore, the XRD patterns of calcined samples after heat treatment at 400 °C are displayed in Figure 3. It can be clearly seen that the crystal structure of the samples exhibits typical periclase phase of MgO and/or zincite phase of ZnO, with diffraction peaks at 37.0°, 43.0°, 62.3° (JCPD 01-077-2364) and 31.8°, 34.4°, 36.3°, 47.6°, 56.6°, 62.9°, 68.0° (JCPD 01-089-0510), respectively. When 5 wt.% of Mg^2+^ cations were substituted by Zn^2+^ ions (Au_25_/Zn_0.05_MgAl-400, Figure 3b), the structure of the catalyst was quite similar to the binary Au_25_/MgAl-400. When the replacement increased to 35.6 wt.% (Au_25_/Zn_0.33_MgAl-400, Figure 3c), both MgO and ZnO phase appears in the patterns. Further increasing the replacement of Zn^2+^ to 70.9 wt.% (Au_25_/Zn_3_MgAl-400, Figure 3d), the phase of ZnO dominates the structure. It implies that the addition of Zn^2+^ ions strongly affected the ordered structure of catalysts. In addition, no diffraction peaks attributed to face-centered cubic gold were observed in all heat-treated samples, indicating that the gold particles were highly dispersed and not aggregated under high temperature calcination.

### 2.3. Particle Size of Gold 

**Figure 4:**
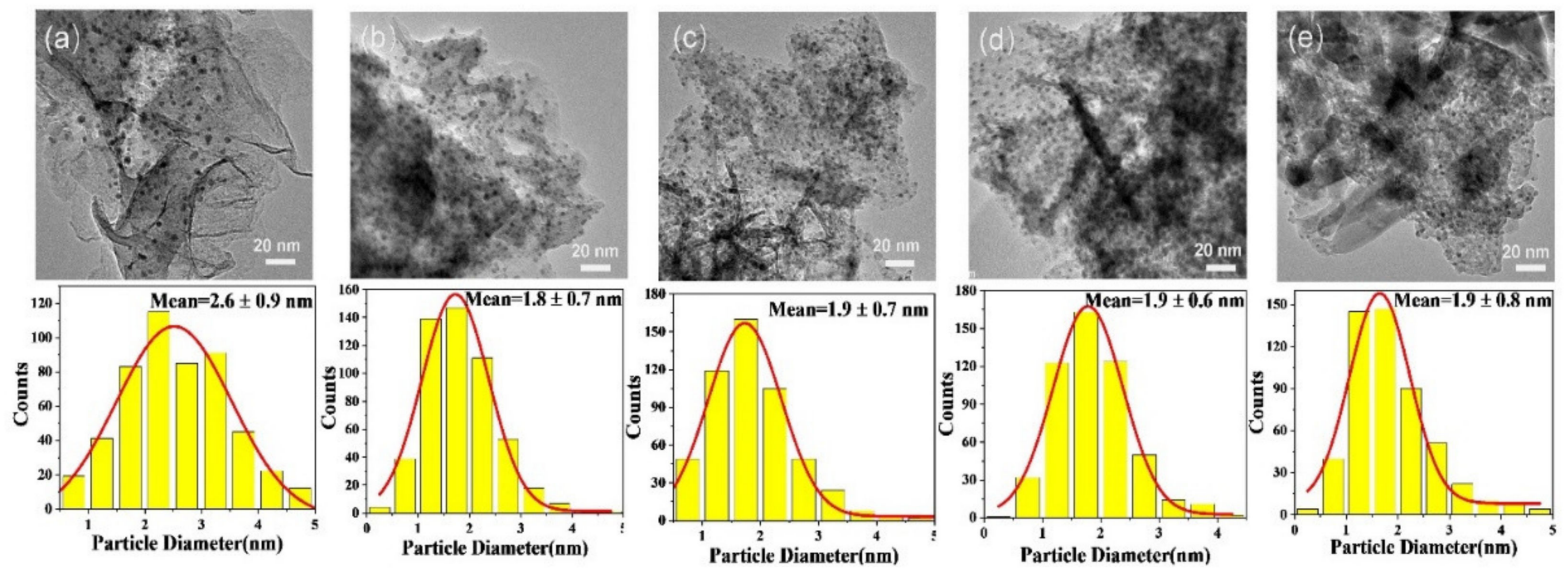
TEM images of Zn-Mg-Al mixed oxides-supported gold catalysts: (**a**) Au_25_/MgAl-400; (**b**) Au_25_/Zn_0.05_MgAl-400; (**c**) Au_25_/Zn_0.33_MgAl-400; (**d**) Au_25_/Zn_3_MgAl-400; (**e**) Au_25_/ZnAl-400.

To further observe the dispersion of gold particles and examine the average particle sizes, transmission electron microscopy (TEM) was performed over the above catalysts. The TEM images and size distribution histograms are displayed in Figure 4. From the photos, the gold particles in all samples are almost highly dispersed, with average particle sizes of 2.6 ± 0.9, 1.8 ± 0.7, 1.9 ± 0.7, 1.9 ± 0.6, 1.9 ± 0.8 nm for the Au_25_/MgAl-400 (Figure 4a), Au_25_/Zn_0.05_MgAl-400 (Figure 4b), Au_25_/Zn_0.33_MgAl-400 (Figure 4c), Au_25_/Zn_3_MgAl-400 (Figure 4d) and Au_25_/ZnAl-400 (Figure 4e) catalysts, respectively. Obviously, the addition of Zn^2+^ ions greatly reduced the particle sizes of gold from 2.6 nm to 1.8 nm, and facilitated the dispersion, which probably derived from the strong interaction between gold with Zn-contained carrier [30,37].

### 2.4. Electronic Property of the Catalysts 

**Figure 5:**
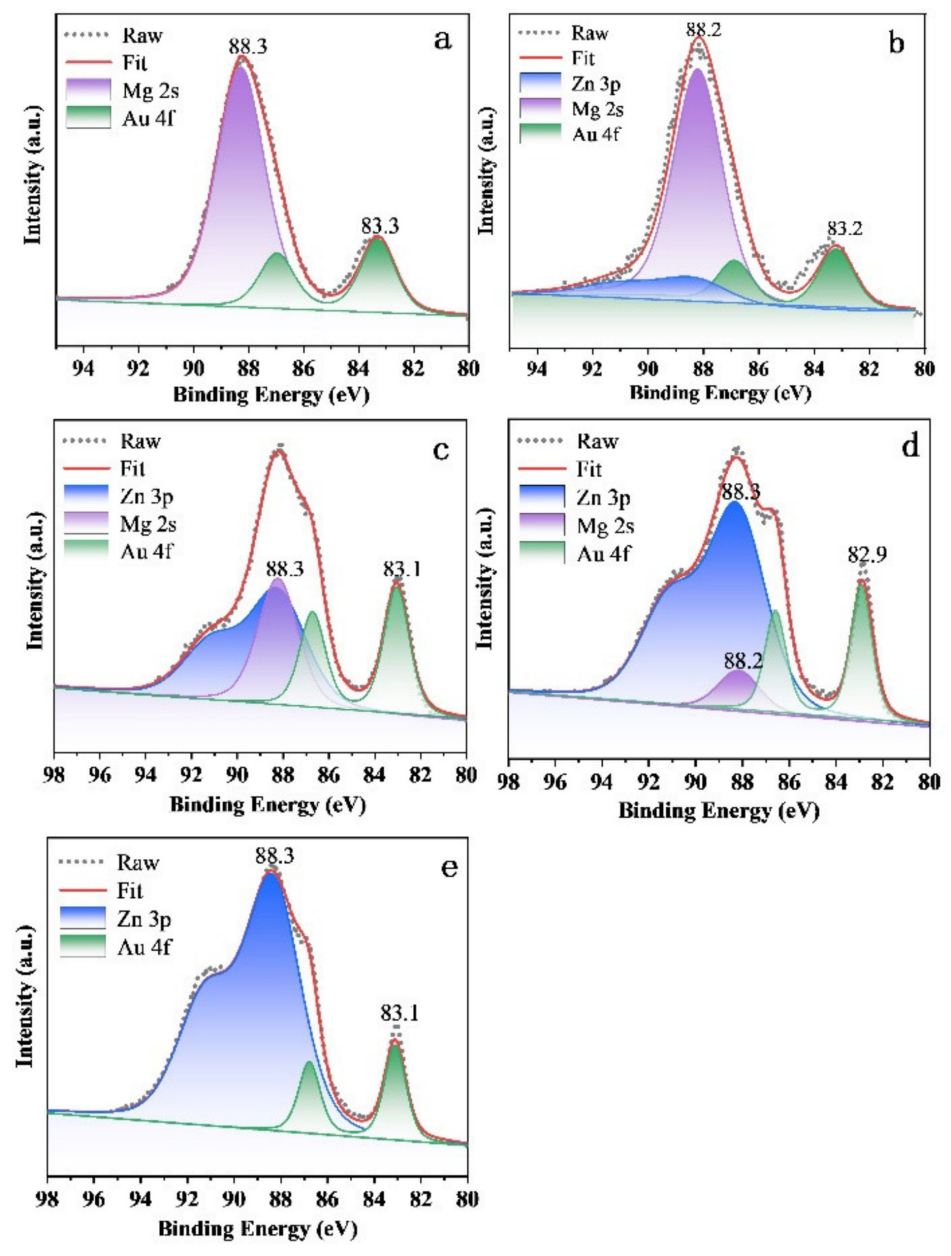
XPS Spectra of Au 4f-Zn 3p-Mg 2s of Zn-Mg-Al oxides-supported gold catalysts: (**a**) Au_25_/MgAl-400; (**b**) Au_25_/Zn_0.05_MgAl-400; (**c**) Au_25_/Zn_0.33_MgAl-400; (**d**) Au_25_/Zn_3_MgAl-400; (**e**) Au_25_/ZnAl-400.

To study the chemical state of elements in different samples, X-ray photoelectron spectroscopy (XPS) was performed on C1s, O1s, Au 4f, Zn 3p and Mg 2s, as shown in Figures S2 and S3, and Figure 5. The binding energy of each element was calibrated by referencing them to the energy of the C1s peak at 284.6 eV (Figure S2). All catalysts showed distinguished peaks around at 83.3 eV and 87.0 eV, respectively, which could be assigned to the Au 4f 7/2 and Au 4f 5/2 lines of metallic Au (Figure 5) [38]. The peak located at 88.2/88.3 eV is assigned to the peaks of Mg 2s and/or Zn 3p, which is identified by the purple and blue bands. Obviously, with the increase in Zn^2+^ ions, the intensity of peaks ascribed to Zn 3p increase, but the peaks of Mg 2s decrease correspondingly. Of note, a slight shift to low binding energy of Au was observed over the Au_25_/Zn_0.05_MgAl-400 (83.2 eV, Figure 5b), Au_25_/Zn_0.33_MgAl-400 (83.1 eV, Figure 5c), Au_25_/Zn_3_MgAl-400 (82.9 eV, Figure 5d) and Au_25_/ZnAl-400 (83.1 eV, Figure 5e) catalysts, indicating there might be a strong interaction between gold with Zn-contained carrier. This was consistent with the results of TEM, which shows the reduction of sizes originated from the interaction of gold with the Zn-contained carrier. The O 1s XPS spectra of different gold catalysts are presented in Figure S3, which shows two peaks. One peak located at 529.7~530.2 eV was attributed to the lattice oxygen connected with Mg^2+^ and/or Zn^2+^ [39], whereas the other peak located at 531.3~531.6 eV was assigned to hydroxide and adsorbed water [39,40], which originated from the interlaminar anions of hydrotalcites.

### 2.5. Basicity of the Catalysts 

**Figure 6:**
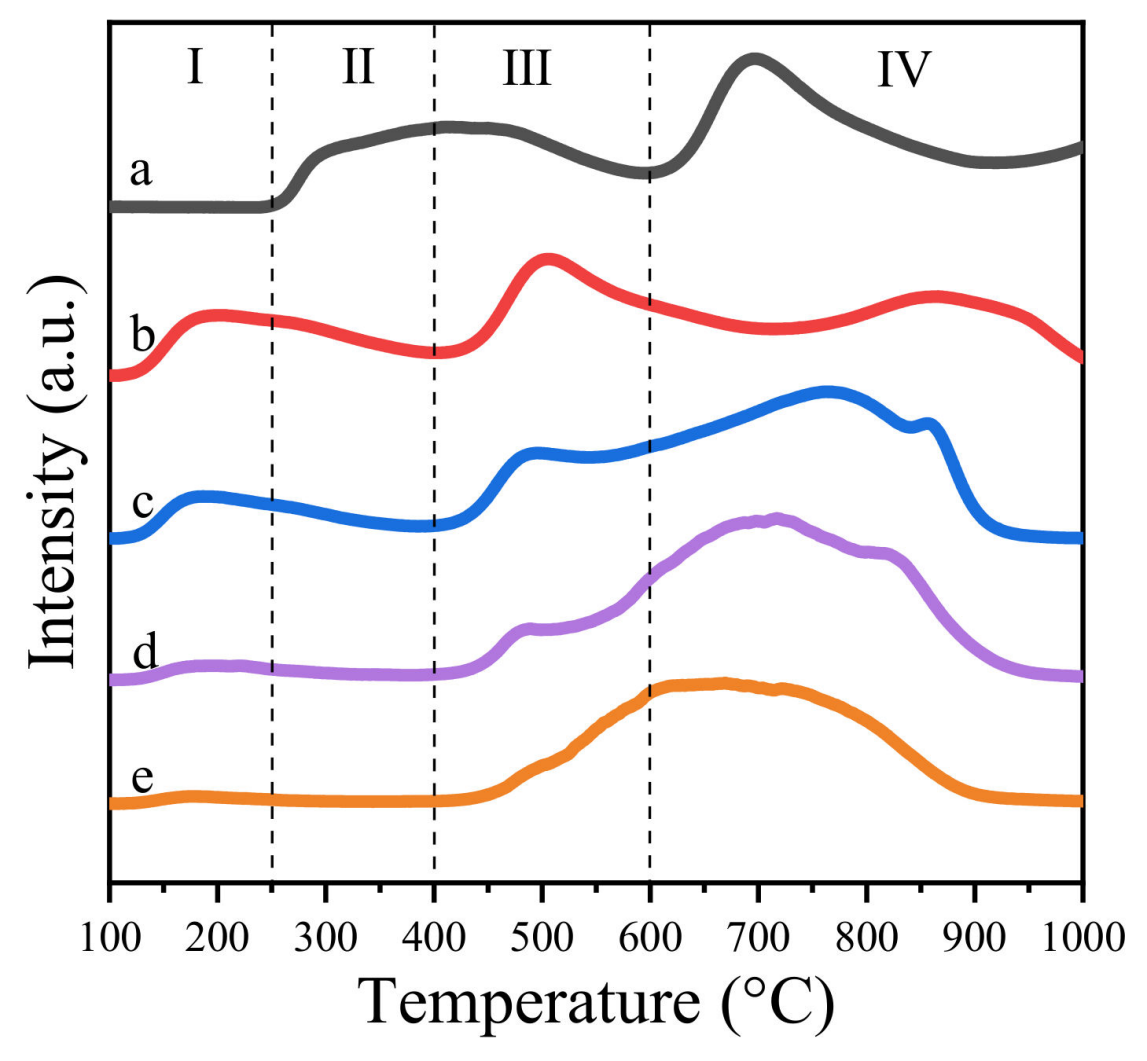
CO_2_-TPD profiles of Zn-Mg-Al mixed oxides-supported gold catalysts: (**a**) Au_25_/MgAl-400; (**b**) Au_25_/Zn_0.05_MgAl-400; (**c**) Au_25_/Zn_0.33_MgAl-400; (**d**) Au_25_/Zn_3_MgAl-400; (**e**) Au_25_/ZnAl-400.

Mg-Al hydrotalcite or its derived oxide is a well-known solid base, which is widely used as a catalyst or support [19,20,21,22]. In the literature, the basicity of the catalyst was reported to facilitate the oxidative esterification of aldols [41,42]. Our group also reported that the strong basic sites benefit the formation of hemiacetal intermediate, which contributes to the synthesis of the final product [31]. Hence, in this work, CO_2_-TPD was utilized to determine the intensity of basic sites of Zn-Mg-Al mixed oxides-supported gold catalysts. The amounts of basic sites were quantitatively measured by CO_2_-pulse-adsorption experiments. The results are summarized in Table 1. It is clear that the basicity of catalysts sharply decreased from 158 μmol·g^−1^ to 23 μmol·g^−1^ with the increase in Zn^2+^ ions. This implies the basicity of catalysts mainly derived from the MgO phase in the composite oxides [21]. Furthermore, according to the desorption temperature of carbon dioxide, the basic sites could be roughly classified into four types, namely, weak basic sites (<250 °C), medium basic sites (250–400 °C), strong basic sites (400–620 °C) and super strong basic sites (>620 °C) [41,43]. Generally, the weak basic sites were caused by hydroxide groups [44]. The medium basic sites were attributed to M^2+^-O^2-^ pairs and the strong basic sites were assigned to lattice oxygen [44]. As displayed in Figure 6, the amounts of weak basic sites increased with the increase of Zn, but the medium basic sites and strong basic sites reduced accordingly. 

### 2.6. Catalytic Performances 

**Scheme 1:**
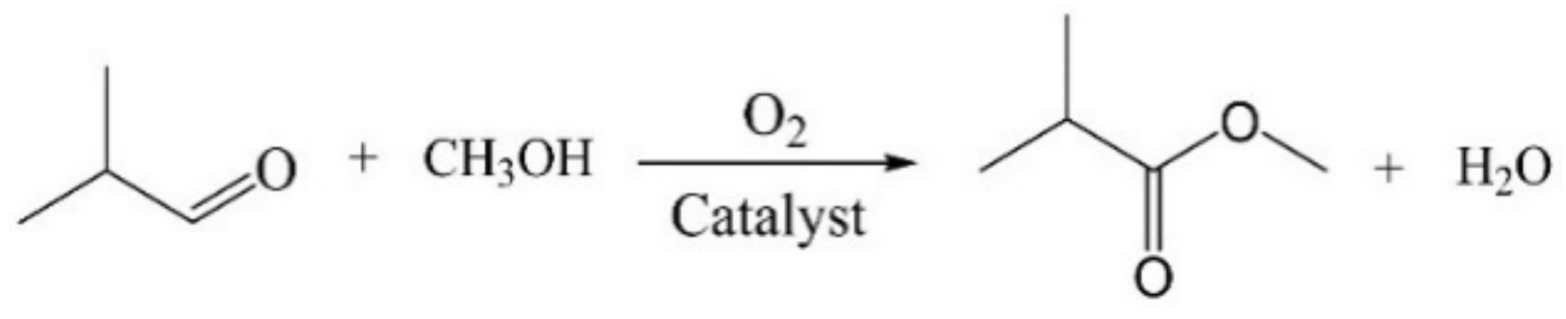
Reaction pathway of isobutyraldehyde with MeOH to form methyl isobutyrate.

**Figure 7:**
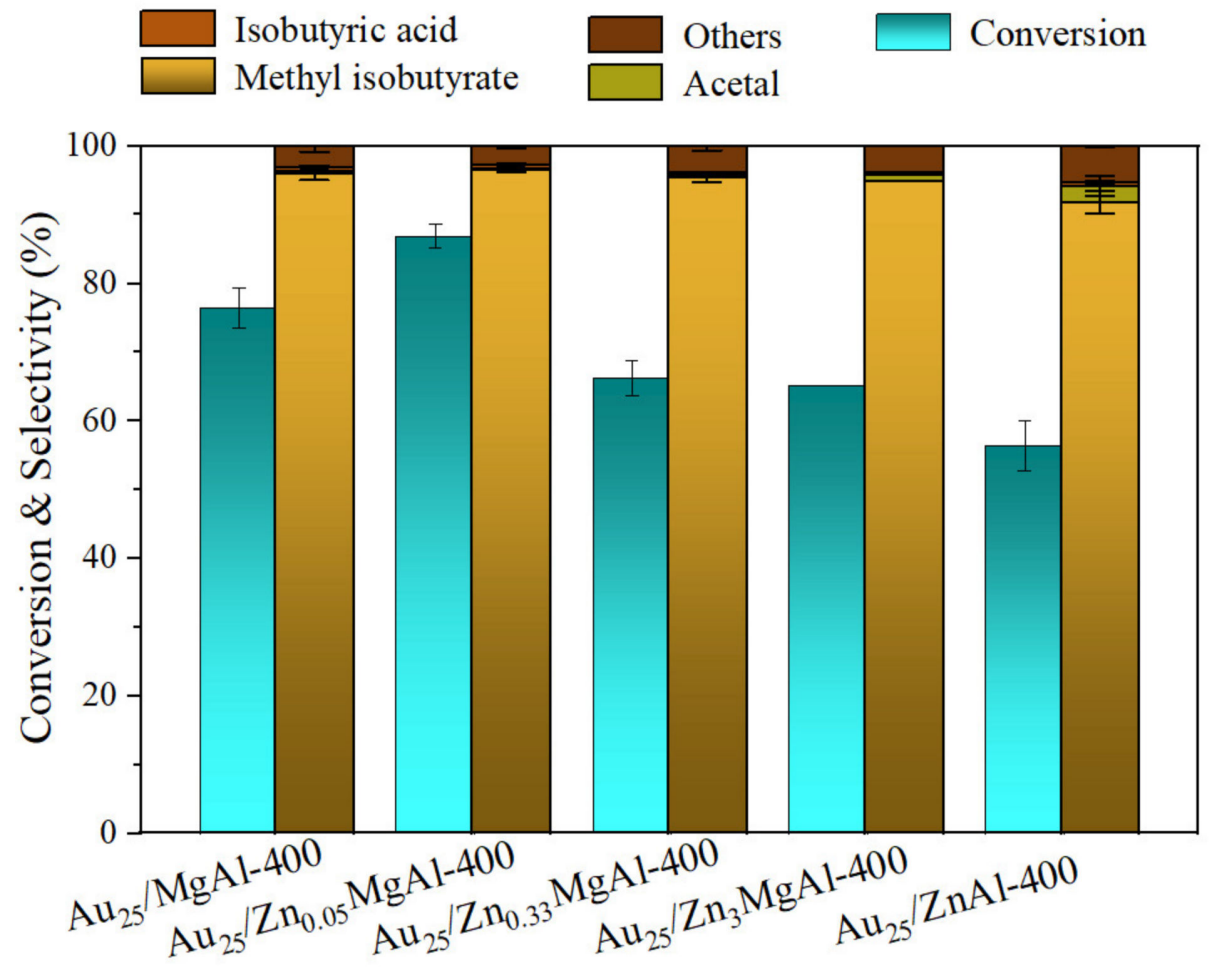
Catalytic performances of Zn-Mg-Al mixed oxides-supported gold catalysts for synthesis of methyl isobutyrate with error bar. Reaction conditions: catalyst: 40 mg; isobutyl aldehyde: 5 mmol; methanol: 5 mL; reaction temperature: 80 °C; pressure of O_2_: 4 atm; reaction time: 2 h.

The catalytic performances of above Zn-Mg-Al mixed oxides-supported gold catalysts were investigated in direct oxidative esterification of isobutyraldehyde with methanol for synthesis of methyl isobutyrate (Scheme 1). The results are displayed in Table 3 and Figure S4. From the results, the Au_25_/MgAl-400 catalyst showed good performance with conversion of 78.5% and selectivity of 96.8% (Table 3, entry 1). When 5% of Zn^2+^ ions were added into the Mg-Al binary system, the conversion of isobutyraldehyde increased to 88.6%, with the selectivity of 96.6%. Meanwhile, the turnover frequency (TOF) of gold catalysts increased from 1499 to 1933 h^−1^ (Table 3, entry 2), suggesting that the catalytic performance could be well improved by the addition of Zn. However, further increase in the content of Zn^2+^ ions would not enhance the catalytic performances. On the contrary, the activity and selectivity would reduce consecutively. For example, over the Au_25_/Zn_0.33_MgAl-400 catalyst, the conversion and selectivity are 68.0% and 95.9%, respectively. Over the Au_25_/Zn_3_MgAl-400 catalyst, the conversion and selectivity decreased to 65.2% and 95.0%. On the Au_25_/ZnAl-400 catalyst, the conversion reduced to 59.0% and the selectivity decreased to 93.0%. This meant the optimum addition amount of Zn follows the volcanic curve, and only small amount of Zn promotes the catalytic reactivity. To further affirm the result, we also performed the reaction under the same conditions three times and plotted the histogram with error bar, as displayed in Figure 7. It is clear that the Au_25_/Zn_0.05_MgAl-400 catalyst indeed shows the best performance. 

To further study the evolution of product distributions during the reaction process, dynamic experiments were conducted over the Au_25_/Zn_x_MgAl-400 catalysts. The yields of methyl isobutyrate and by-products of acetal and isobutyric acid with reaction time are plotted in Figure S4. From the curves, the trends on product distributions over different catalysts were somewhat different. In the process of reaction, methyl isobutyrate was formed as the sacrifice of isobutyraldehyde. At the same time, acetal, isobutyric acid and other ester compounds appeared as the by-products. Of note, the content of acetal and isobutyric acid are more with the Au_25_/ZnAl-400 catalyst than the others, implying the transformation of isobutyraldehyde to its final target product is harder on the Au_25_/ZnAl-400 catalyst than that of the Au_25_/Zn_x_MgAl-400 catalysts.

## 3. Discussion

According to our previous characterizations, the small amounts of Zn^2+^ ions (~5 wt.%) would lead to the reduction of gold particle size to below 2 nm (Figure 4) and enhance the interaction between gold with the support (Figure 5). Hence, we have a reason to believe that the improved activity is connected with the decreased sizes ascribed to the addition of Zn. In fact, many investigations have demonstrated that the activity of gold catalyst is markedly dependent on the particle size [45,46], since small gold particles possess more coordinated-unsaturated gold species, which supply more adsorption sites for reactants [46]. However, based on the results of catalytic performances, further increasing the content of Zn^2+^ ions would decrease the activity, even though the particle size of gold is still below 2 nm. Thus, this strongly suggests the size of gold was not the only factor that influences the catalytic performance.

Previously, we have proposed that the basicity of catalysts is critical for oxidative esterification [31]. Various works also suggest that the basic sites can accelerate the formation of hemiacetal intermediate, thus contributing to the formation of target esters [47,48]. To examine the basic sites of our catalysts, CO_2_-TPD experiments were then carried out on the above catalysts. From the results, it is clear to see the total basicity of catalysts decreased gradually with the increase of Zn^2+^ ions (Table 1). Meanwhile, the intensity of strong basic sites decreased correspondingly (Figure 6). Based on the previous literature, the strong basic sites are proposed to be the key sites for enhancement of the intermediate [47,49]. Thus, with the decrease of intensity assigned to strong basic sites, the catalytic performances would be decreased. This is consistent with our result. Therefore, the activity of Zn-Mg-Al mixed oxides-supported gold catalysts were supposed to be sectionally influenced by the particle size of gold and the basicity of supports. 

Furthermore, to clarify the effect of loading gold particles, comparison experiments were conducted on Zn_0.05_MgAl-400 catalyst or with N_2_ as the source gas (Table 3, entry 7, 6). Both situations give no products after reaction under the same conditions, suggesting that the gold particles were the critical components for oxidative esterification. Our previous work demonstrated that a calcination temperature above 300 °C was sufficient to remove most of the protective ligands [30], so that bare gold particles could be exposed on the surface of catalyst. Besides, it has been reported that the gold clusters below 2 nm could be active for dissociation of the molecular oxygen [50,51]. Hence, with respect to this work, we speculate that the role of gold is probably activating and dissociating oxygen so the oxidative esterification could be progressed successfully. 

Considering the effect of basic sites for transformation of isobutyl aldehyde and the large role of dissociation of oxygen on the gold particles, the active center is believed to be located at the interface between metallic gold with the support. Therefore, the reaction mechanism over the above catalysts could be proposed based on the results and the literature [31,49,52]. That is, oxygen was dissociated into the adsorbed atoms on gold particles and reacts with methanol to form methoxy groups [46,50]. Then, the isobutyl aldehyde molecules were oxidized by atomic oxygen to form isobutyric acid or underwent nucleophilic attack by methoxy groups to form the surface hemiacetal intermediate. Later, during the oxidation of intermediate hemiacetal, the hemiacetal transforms to the target ester via β-H elimination with the assistant of atomic oxygen or forms the acetal by-product by reacting with methoxy groups.

## 4. Materials and Methods

### 4.1. Materials

Sodium hydroxide (NaOH, AR), sodium carbonate (Na_2_CO_3_, AR), hydrogen tetrachloroaurate hydrate (HAuCl_4_·3H_2_O), methanol (AR) and aluminum nitrate hydrate (Al(NO_3_)_3_·9H_2_O), 99%) were purchased from Sinopharm. Sodium borohydride (NaBH_4_, 97%) and magnesium nitrate hexahydrate (Mg(NO_3_)_3_·6H_2_O), 99%) were purchased from Shanghai Lingfeng Chemical Reagent Company. Isobutyl aldehyde (98%), cysteine (99%), methyl isobutyrate (99%), ortho-xylene (CP) and zinc nitrate hydrate (Zn(NO_3_)_2_·6H_2_O), 99%) were purchased from Aladdin Industrial Corporation. All chemicals were used directly without any purification. All glassware was washed with Aqua Regia and rinsed with ethanol and ultrapure water (18.2 MΩ).

### 4.2. Catalyst Preparation

The Zn-Mg-Al hydrotalcites (HTs) with different Zn^2+^/Mg^2+^ molar ratios were prepared by a co-precipitation method. Typically, specific amounts of Zn(NO_3_)_2_·6H_2_O (0, 0.03, 0.2, 0.42, 0.63 mol), Mg(NO_3_)_2_·6H_2_O (0.63, 0.6, 0.42, 0.2, 0 mol) and Al(NO_3_)_3_·9H_2_O (0.21 mol) were mixed with 200 mL of ultrapure water to obtain a solution (denoted as solution A). Na_2_CO_3_ (0.113 mol) and NaOH (0.438 mol) were mixed with 200 mL of ultrapure water in a 500 mL round-bottom flask to obtain solution B. Under vigorous stirring in a water bath at 70 °C, solution A was pumped into solution B very slowly. Then, the obtained mixture was aged at 70 °C for about 24 h with constant stirring. Following filtering, washing and drying overnight, the Zn-Mg-Al HTs were obtained, which were denoted as MgAl-HT; Zn_0.05_MgAl-HT; Zn_0.33_MgAl-HT; Zn_3_MgAl-HT and ZnAl-HT (0.05, 0.33 and 3 denote the molar ratio of Zn^2+^/Mg^2+^).

Zn-Mg-Al mixed oxides-supported gold catalysts were prepared as follows. Firstly, gold clusters protected by thiolate-ligands (cysteine) were prepared by a NaBH_4_ reduction method according to our previous work [30]. Typically, 1.485 mL of HAuCl_4_ (19.12 g_Au_/L) was dissolved in 25 mL of ultrapure water under vigorous stirring for 5 min. Then, 37.5 mL of cysteine solution (5.61 mM) and 7.5 mL of 1 M NaOH solution were successively added into the solution. After about 1 h, excessive and fresh prepared NaBH_4_ solution was poured into the solution quickly and we continued to stir for 3 h. UV-Vis spectra were analyzed to define the products as Au_25_ nanoclusters. Then, about 2.5 g of powder of support was added into the aqueous solution of Au_25_ clusters. Then, 2 h later, the resulting mixture was filtered, washed and dried to obtain the precursor of the catalyst. Before catalytic testing, the precursors of the catalysts were calcined at 400 °C for 120 min, which were denoted as Au_25_/MgAl-400; Au_25_/Zn_0.05_MgAl-400; Au_25_/Zn_0.33_MgAl-400; Au_25_/Zn_3_MgAl-400 and Au_25_/ZnAl-400.

### 4.3. Catalyst Characterization

The actual loadings of gold in all of the catalysts were determined by inductively coupled plasma atomic emission spectroscopy (ICP-AES) on an IRIS Intrepid II XSP instrument (Thermo Electron Corporation, Waltham, Massachusetts, USA). UV-Vis absorption spectroscopy (EVO300, Waltham, Massachusetts, USA) was utilized to precisely define the atomic Au_25_ nanoclusters, with water as the reference. The X-ray diffraction (XRD) analysis was conducted on a PW3040/60 X’Pert PRO (PANalytical, Almelo, The Netherlands) diffractometer, with a Cu Kα radiation source (λ = 0.15432 nm) in a scanning angle (2θ) range of 10–90°. The N_2_-physical adsorption–desorption was measured at 77 K on a Quantachrome NT3LX-2 instrument after degassing the samples at 300 °C for 120 min. The pore size distribution and the specific surface area were calculated by the BJH and BET methods. The high-resolution transmission electron microscopy (HRTEM) and TEM were recorded on a JEM-2100F microscope at 200 kV. The X-ray photoelectron spectra (XPS) were conducted on an ESCLALAB 250Xi X-ray photoelectron spectrometer equipped with a monochromatic Al and double anode Al/Mg target. The binding energy was calibrated using the C 1s peak (284.6 eV) as the reference. The temperature-programmed desorption of carbon dioxide (CO_2_-TPD) experiments was studied on a Micromeritics Autochem II 2920 chemisorber equipped with a thermal conductivity detector and mass spectrometry. Firstly, about 100 mg of the catalysts was pretreated in a U-type quartz tube reactor with a He gas stream (50 cm^3^/min) at 400 °C for 30 min. After the temperature decreased to 100 °C, the catalysts were saturated with 10%CO_2_/He flow (30 cm^3^/min) for 15 min. Then, the samples were purged in helium at 100 °C for 30 min and waited for baseline balance. Finally, the CO_2_-TPD profile of the sample was recorded by increasing the temperature from 100 °C to 1000 °C at a heating rate of 10 °C/min under He flow.

### 4.4. Catalytic Test

Catalytic evaluation of one step oxidative esterification for synthesis of methyl isobutyrate from isobutyraldehyde and methanol was carried out in stainless steel autoclave equipped with magnetic stirring and pressure gauge. Typically, certain amounts of catalysts and the mixture of isobutyraldehyde (1 M) and methanol (5 mL) were introduced into the reactor. After sealing, the air in the autoclave was replaced with oxygen six times and then pressurized to 4 atm. Before the reaction, the reactants were gradually heated to 353 K in a water bath and initiated to stir. When the reaction finished, the reactor was placed in an ice bath immediately to terminate the reaction. The products were identified by GC−MS (Agilent 5977B MSD GC/MS) and analyzed quantitatively by GC (Agilent 7890 A) with a flame ionization detector (FID) and a HP-5 column (30 m, 0.25 mm inner diameter).

## 5. Conclusions

A series of Zn-Mg-Al hydrotalcites and derived mixed oxides-supported gold catalysts with different Zn^2+^/Mg^2+^ molar ratios were prepared for one-step oxidative esterification of isobutyraldehyde with methanol to form methyl isobutyrate. Adding a small amount of Zn^2+^ ions (~5 wt.%) provoked a remarkable modification of catalytic performance for the binary Mg-Al system, which enhanced the activity of catalysts from 78.5% to 88.6% and TOF values from 1499 h^−1^ to 1933 h^−1^. Characterizations of structure and electronic properties demonstrated the addition of Zn not only reduced the particle size of gold but also enhanced the interaction between gold with the support. Furthermore, the catalytic performance was also highly dependent on the basicity of the catalyst. Based on a comparison experiment, the active center was believed to be located at the interface between metallic gold with the support, in which basicity plays a big role in transformation of the intermediates. This work provides deep insights into one-step oxidation esterification of aldehydes and alcohols over ternary system and supported gold catalysts.

## Figures and Tables

**Table 1:** Textural properties of Zn-Mg-Al mixed oxides-supported gold catalysts.

Entry	Catalysts	Loadings of Au (%) ^a^	S_BET_ (m^2^g^−1^) ^b^	Volume (cm^3^g^−1^) ^b^	D_pore_ (nm) ^b^	Particle Size (nm) ^c^	Total Basicity (μmol g^−1^) ^d^
1	Au_25_/MgAl-400	1.52	221.1	0.51	0.57	2.6	158
2	Au_25_/Zn_0.05_MgAl-400	1.52	242.0	0.51	0.57	1.8	111
3	Au_25_/Zn_0.33_MgAl-400	1.52	132.4	0.37	0.56	1.9	81
4	Au_25_/Zn_3_MgAl-400	1.47	113.9	0.35	0.56	1.9	23
5	Au_25_/ZnAl-400	1.50	91.1	0.24	0.56	1.9	29

^a^: The actual loadings of gold were evaluated by ICP-AES measurement. ^b^: Specific surface area (S_BET_), total pore volume and average pore size were calculated from N_2_ physical adsorption–desorption. S_BET_ were calculated from BET equation at P/P_0_ range of 0.05-0.3. Total pore volumes were calculated at P/P_0_ = 0.98. Pore sizes were calculated from the adsorption branch of the isotherm-BJH. ^c^: The mean particle sizes were calculated from TEM measurement. ^d^: Total basicity was calculated from CO_2_-TPD experiments.

**Table 2:** Crystallographic parameters calculated for Zn-Mg-Al HTs supported gold nanoclusters.

Entry	Catalysts	d_(003)_ (nm)	d_(110)_ (nm)	*a* (nm) ^a^	*c* (nm) ^b^	Crystallite Size (nm) ^c^	
						(003)	(110)
1	Au_25_/MgAl-400	0.772	0.153	0.306	2.316	18	26
2	Au_25_/Zn_0.05_MgAl-400	0.769	0.153	0.306	2.307	11	22
3	Au_25_/Zn_0.33_MgAl-400	0.763	0.153	0.306	2.289	9	22
4	Au_25_/Zn_3_MgAl-400	0.760	0.153	0.306	2.280	17	31
5	Au_25_/ZnAl-400	0.755	0.154	0.308	2.265	33	30

^a^: Lattice parameter: *a* = 2d_110_. ^b^: Lattice parameter: *c* = 3d_003_. ^c^: Average crystallite size: calculated using the Scherrer equation.

**Table 3:** Catalytic performances of Zn-Mg-Al mixed oxides-supported gold catalysts for synthesis of methyl isobutyrate from isobutyraldehyde and methanol.

Entry	Catalysts	Conversion% ^a^	Selectivity % ^a^				TOF (h^−1^) ^c^
			Methyl Isobutyrate	Isobutyric Acid	Acetal	Others	
1	Au_25_/MgAl-400	78.5	96.8	0.54	0.27	2.43	1499
2	Au_25_/Zn_0.05_MgAl-400	88.6	96.6	0.73	0.20	2.45	1933
3	Au_25_/Zn_0.33_MgAl-400	68.0	95.9	0.29	0.49	3.32	1790
4	Au_25_/Zn_3_MgAl-400	65.2	95.0	0.38	0.74	3.93	1713
5	Au_25_/ZnAl-400	59.0	93.0	0.23	1.28	5.48	1252
6	Au_25_/Zn_0.05_MgAl-400 ^b^	2.12	Trace	-	-	-	-
7	Zn_0.05_MgAl-400	5.26	Trace	-	-	-	-

Reaction conditions: Amounts of catalyst: 40 mg; isobutyl aldehyde: 5 mmol; methanol: 5 mL; o-xylene (interior standard): 1 mmol; reaction temperature: 80 °C; pressure of O_2_: 4 atm; reaction time: 2 h. ^a^: Conversion and selectivity were calculated by the results of gas chromatography; ^b^: O_2_ was replaced by N_2_ (4 atm); ^c^: TOF was calculated by moles of converted aldehyde per mole of gold per hour, the reaction rate was calculated below 20% conversion.

## Data Availability

Data is available upon request from the corresponding authors.

## Supplementary Materials

The following are available online at https://www.mdpi.com/article/10.3390/ijms22168668/s1.
