# Supplementary material for: Zn Promoted Mg-Al Mixed Oxides-Supported Gold Nanoclusters for Direct Oxidative Esterification of Aldehyde to Ester
Source: Int J Mol Sci. 2021 Aug 12;22(16):8668. doi: 10.3390/ijms22168668 (PMC8395456; doi:10.3390/ijms22168668)
Supplement: Supplementary file 1 [file ijms-22-08668-s001.zip › ijms-1301850-supplementary.pdf]

Zn promoted Mg-Al mixed oxides supported gold nanoclusters for direct oxidative esterification of aldehyde to ester

Jie Li,[a] Shiyi Wang,[a] Huayin Li,[a] Yuan Tan,[a] Yunjie Ding *[a,b,c]*

[a] *Hangzhou Institute of Advanced studies, Zhejiang Normal University, Hangzhou 311231, China.*

[b] *Dalian National Laboratory for Clean Energy, Dalian Institute of Chemical Physics, Chinese Academy of Sciences, Dalian 116023, China.*

[c] *The State Key Laboratory of Catalysis, Dalian Institute of Chemical Physics, Chinese Academy of Sciences, Dalian 116023, China.*

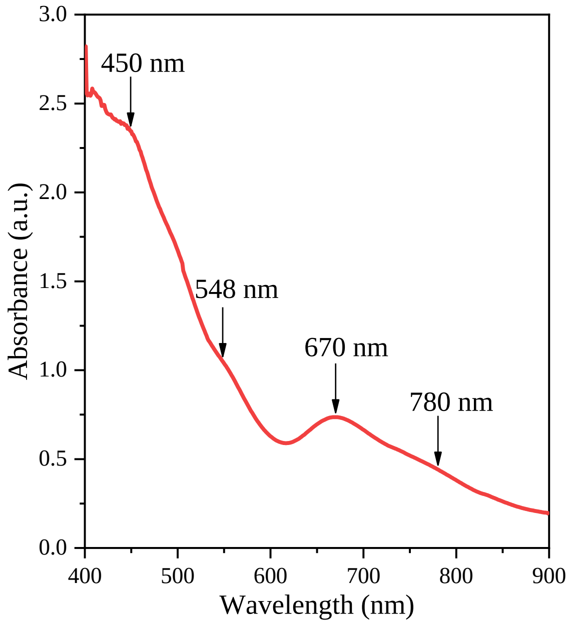

Figure S1. UV-visible spectra of just-synthesized Au_{25} nanoclusters.

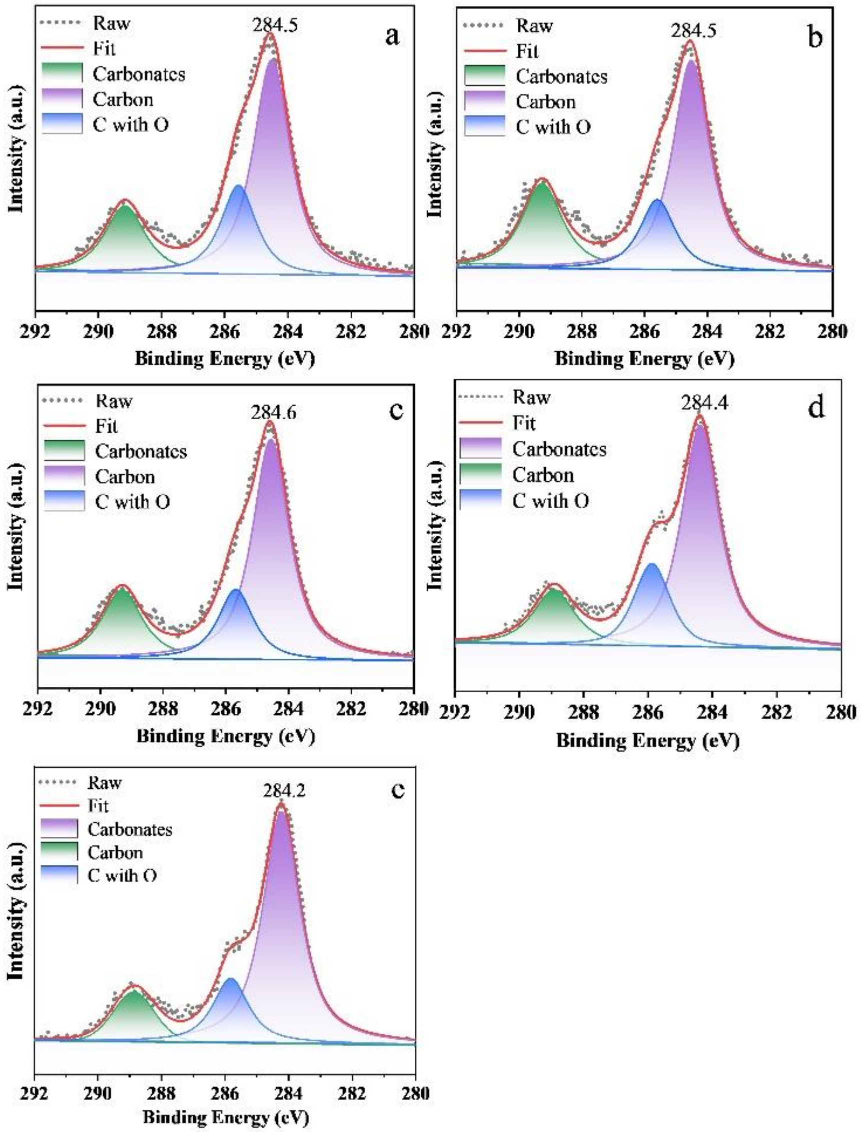

Figure S2. XPS spectra of C 1s of Zn-Mg-Al mixed oxides supported gold catalysts: (a) Au_{25}/MgAl-400; (b) Au_{25}/$Zn_{0.03}$MgAl-400; (c) Au_{25}/$Zn_{0.33}$MgAl-400-; (d) Au_{25}/Zn_{3}MgAl-400; (e) Au_{25}/ZnAl-400.

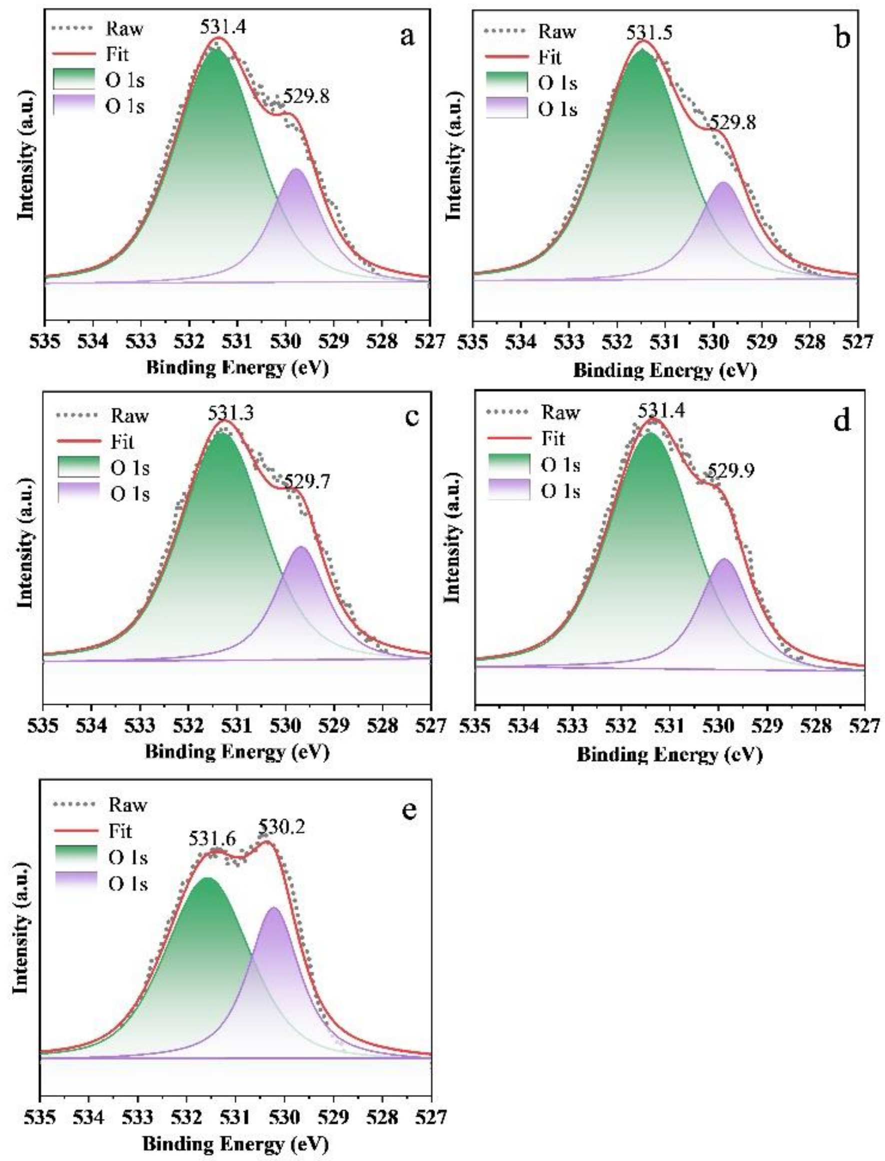

Figure S3. XPS spectra of O 1s of Zn-Mg-Al mixed oxides supported gold catalysts: (a) Au_{25}/MgAl-400; (b) $Au_{25}/Zn_{0.03}$MgAl-400; (c) $Au_{25}/Zn_{0.33}$MgAl-400-; (d) Au_{25}/Zn_{3}MgAl-400; (e) Au_{25}/ZnAl-400.

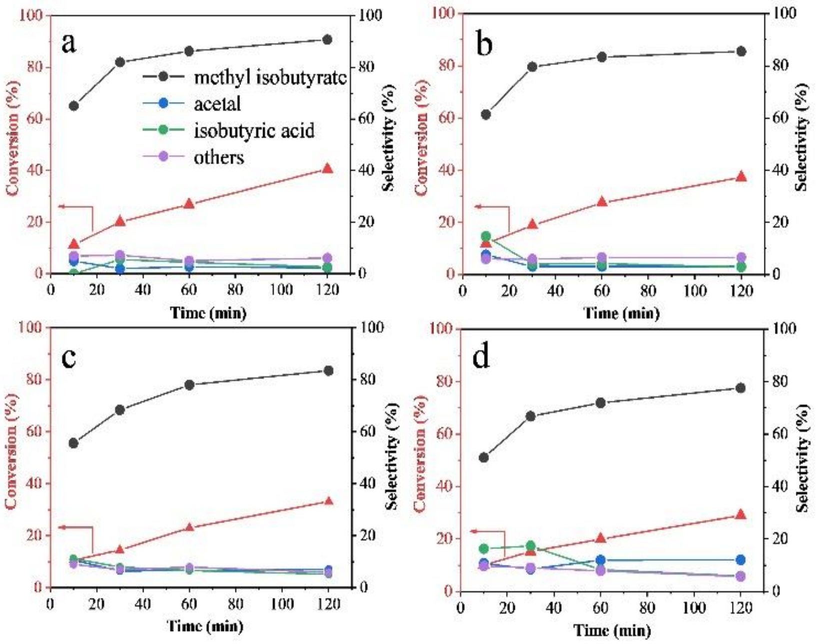

Figure S4. The products distribution of reaction with time courses: (a) Au_{25}/MgAl-400; (b) $Au_{25}/Zn_{0.33}MgAl$-400; (c) Au_{25}/Zn_3MgAl-400; (d) Au_{25}/ZnAl-400. Reaction conditions: 5 mmol of isobutyl aldehyde and 5 mL of methanol; Catalyst: 30 mg; Pressure of O_2: 4 atm; Reaction temperature: 80 °C.
